# Subtyping Cutaneous Melanoma Matters

**DOI:** 10.1093/jncics/pkaa097

**Published:** 2020-10-23

**Authors:** Mary-Ann El Sharouni, Paul Johannes van Diest, Arjen Joost Witkamp, Vigfús Sigurdsson, Carla Henrica van Gils

**Affiliations:** 1 Department of Dermatology, University Medical Center Utrecht, Utrecht University, Utrecht, the Netherlands; 2 Department of Pathology, University Medical Center Utrecht, Utrecht University, Utrecht, the Netherlands; 3 Department of Surgery, University Medical Center Utrecht, Utrecht University, Utrecht, the Netherlands; 4 Department of Epidemiology, Julius Center for Health Sciences and Primary Care, University Medical Center Utrecht, Utrecht University, Utrecht, the Netherlands

## Abstract

**Background:**

Our aim was to investigate the role of melanoma subtype on survival and focus on the effects stratified by Breslow thickness and ulceration status.

**Methods:**

Patients with cutaneous melanoma stage I, II, or III diagnosed between 2000 and 2014 were derived from the Dutch Nationwide Pathology Registry and overall survival data from the Netherlands Cancer Registry. Patients were followed until 2018. Using multivariable Cox proportional hazards models, hazard ratios were calculated for each melanoma subtype, per Breslow thickness category and ulceration status, and adjusted for age, sex, stage, and localization.

**Results:**

A total of 48 361 patients were included: 79.3% had superficial spreading melanoma (SSM), 14.6% nodular melanoma (NM), 5.2% lentigo maligna melanoma, and 0.9% acral lentiginous melanoma (ALM). In the total patient group, using SSM as the reference category, adjusted hazard ratios were 1.06 (95% confidence interval [CI] = 1.01 to 1.12) for NM, 1.02 (95% CI = 0.93 to 1.13) for lentigo maligna melanoma, and 1.26 (95% = CI 1.06 to 1.50) for ALM. Among patients with 1.0 mm or less Breslow thickness and no ulceration, NM showed a twofold increased risk (hazard ratio = 1.96, 95% CI = 1.58 to 2.45) compared with SSM. Compared with 1.0 mm or less SSM without ulceration, the hazard ratio for 1.0 mm or less SSM with ulceration was 1.94 (95% CI = 1.55 to 2.44), and the hazard ratio for 1.0 mm or less NM with ulceration was 3.46 (95% CI = 2.17 to 5.50). NM patients with tumors greater than 1.0 mm did not show worse survival than SSM patients with tumors greater than 1.0 mm.

**Conclusions:**

In this large nationwide study, ALM patients showed worse survival than SSM patients. Among patients with melanomas that were thin (1.0 mm or less), NM subtype patients also showed worse survival than SSM patients.

Melanoma can be classified into 4 major histologic subtypes: superficial spreading melanoma (SSM), nodular melanoma (NM), lentigo maligna melanoma (LMM), and acral lentiginous melanoma (ALM) (1). SSM is the most common subtype (70%) and usually presents as a flat, slowly growing lesion (2). NM accounts for 20% of all melanomas. As the name suggests, it grows as a nodule, which may be pigmented or amelanotic. NM tends to have a faster growth rate than SSM (3). LMM represent 5%-10% of all melanomas (4) and are mostly diagnosed as large, flat macules on the face in older patients. ALM, by definition, involves the acral sites (palms and soles). It is the most common type of melanoma in the Asian population (5) but is rare (1%-2%) in Western populations (4-6).

Apart from clinical and histological differences, recent studies have shown that there are genetic differences between melanoma subtypes as well. As an example, only a small proportion (16%) of ALM carries a BRAF-mutation compared with up to 66% of SSM (7).

Although current melanoma staging for stage I-III melanoma patients is based on Breslow thickness, ulceration status, and presence of sentinel lymph node metastases (8), it is known that prognosis of patients is also driven by other features, such as age, sex, and anatomic localization (9,10). Regarding histological subtype, there is controversy as to what extent survival differences between melanoma subtypes are driven by the tumor subtype itself or by other well-known correlated prognostic factors, such as a thicker Breslow thickness and more frequent presence of ulceration in some subtypes. The few studies that included a sufficient number of patients to address the prognostic importance of subtype show conflicting results (11-13). However, none of these studies have disentangled the effects of subtype, Breslow thickness, and ulceration status. Therefore, our aim was to investigate the role of melanoma subtypes on survival using nationwide data from the Netherlands. We focused on the 4 major melanoma subtypes in combination with Breslow thickness and ulceration status.

## Methods

### Collection of Data

Data for this retrospective nationwide study were obtained from “PALGA,” the Dutch Nationwide Network and Registry of Histopathology and Cytopathology (14). Since 1991, PALGA has prospectively been collecting data from all pathology laboratories in the Netherlands. All data were encoded and used anonymously. Ethical approval was granted by the board of PALGA.

### Study Population

For this cohort study, pathologic reports of all newly diagnosed invasive melanoma patients in the Netherlands between January 1, 2000, and December 31, 2014, were analyzed. Patients presenting with locoregional (defined as in transit, satellite, or lymph node metastases other than sentinel node biopsy [SLNB]) or distant metastases (stage IV) within 100 days of initial diagnosis were excluded. Patients with noncutaneous melanoma, desmoplastic melanoma, melanoma of unknown primary, and patients without a defined melanoma subtype were excluded. We also excluded patients with multiple primary melanoma, because we previously showed that these patients have worse prognosis (15). Melanoma occurring in children (age <18 years) were excluded as well. For this study, this yielded a dataset of adults with histologically proven invasive, primary, single, cutaneous melanoma diagnosed between 2000 and 2014 in the Netherlands. For each patient, clinical and pathological variables were extracted from the pathology files, including date of diagnosis, age, sex, Breslow thickness in millimeters, T stage, ulceration (present or absent), body site (head and neck, trunk, arms, or legs), melanoma subtype (SSM, NM, LMM, or ALM), and SLNB result (positive, negative, or not performed). Because guidelines do not address the maximum time between primary excision and SLNB, we decided in a multidisciplinary setting to include as SLNB all SLNB performed within 100 days after initial diagnosis, as previously described (16). Patients were categorized as stage I, II, and III according to the 8th edition of the American Joint Committee on Cancer (8). When no SLNB was performed, it was assumed patients were stage I or II. Overall survival data and vital status (dead or alive) were obtained from the Netherlands Cancer Registry hosted by the Comprehensive Cancer Organization of the Netherlands. The Netherlands Cancer Registry is a nationwide, population-based cancer registry with information on vital status and date of death retrieved from the database of deceased persons of the Central Bureau of Genealogy and the municipal demography registries. Follow-up was calculated from date of diagnosis until date of death, the date last known alive, or January 1, 2018, whichever occurred earlier.

### Statistical Analysis

Categorical variables were summarized as numbers and percentages. Continuous variables were summarized as median with interquartile range (IQR) for nonnormally distributed data or mean with SD for normally distributed data. Differences in proportions and medians were analyzed using χ^2^ tests or Mann-Whitney *U* test, respectively. Differences in means were assessed with Student *t* test. Patients were stratified in 4 Breslow thickness strata; 1.0 or less, 1.1-2.0, 2.1-4.0, and greater than 4.0 mm, as well as per ulceration status and stage: I, II, and III. Complete case Cox proportional hazards regression analyses were performed to calculate the main effects of melanoma subtype to estimate hazard ratios (HRs) and 95% confidence intervals (CIs), and time to all-cause death (overall survival) was selected as outcome. Variables selected for multivariable analyses were subtype, Breslow thickness, age, sex, ulceration, localization, and stage. In case of missing ulceration status, ulceration was assumed to be absent. To test if this assumption was valid, we compared the outcomes of a Cox regression model with missing ulceration status as a separate category in a categorical variable with that of a model with missing ulceration status included in the “negative” category. Multiple imputation was not considered, given the pathologist involved in this study (P. J. van Diest) believes from clinical experience that it is plausible that this histopathological parameter is not missing at random but rather because it was not seen during pathological assessment. The missing at random assumption (a condition for multiple imputation) would therefore be too strong. The proportional hazards assumption was examined by plotting a log-minus-log graph for categorical variables. If the lines were parallel, it was assumed that the proportional hazards assumption was not violated. For continuous variables (Breslow thickness and age), Schoenfeld residuals were plotted as a function of time, and a loess curve was fitted. If the curve was horizontal, it was assumed that the proportional hazards assumption was not violated. To assess linearity of continuous variables, Martingale residuals were plotted against time. In case of nonlinearity, continuous variables were categorized. We hypothesized that the effect of melanoma subtype was different for tumors with different Breslow thickness. Hence, we constructed an interaction term of Breslow thickness (categorized as ≤1.0 mm, 1.1-2.0 mm, 2.1-4.0 mm, and >4.0 mm) and ulceration with the 4 subtypes of melanoma and added this to the aforementioned multivariable Cox model. We tested for the presence of statistical interaction by subtracting the deviance (−2*[log likelihood]) from the model with the interaction term from the deviance of the model without the interaction term, evaluating the difference in degrees of freedom and using a χ^2^ distribution to determine the corresponding *P* value. A statistically significant *P* value would indicate that the effect of melanoma subtype is different at different values of Breslow thickness. Finally, we graphically represented the hazard ratios for each melanoma subtype per Breslow thickness category and ulceration. All data were analyzed using SPSS version 26. A 2-sided *P* value of less than .05 was considered statistically significant.

## Results

### Patient Characteristics

A total of 48 361 melanoma patients were included with a female predominance of 56.4% (Table 1). Patients had a mean age at diagnosis of 56.39 years (SD =16.07). The median Breslow thickness was 0.86 mm (IQR = 0.50-1.60 mm). Ulceration was present in 12.5% of patients, and most melanomas were located on the trunk (42.3%). Follow-up data were available in 93.7% of patients, and the median follow-up time was 73.8 months (IQR = 43.5-120.7 months). The median follow-up time among survivors was 82.9 months (IQR = 51.1-129.7 months). The majority of patients were diagnosed with SSM (79.3%), followed by NM (14.6%), LMM (5.2%), and ALM (0.9%). Patients with LMM had a mean age of 71.09 years (SD = 12.37) at the time of diagnosis compared with 54.49 years (SD = 15.44) for SSM patients. The median Breslow thickness varied between 0.60 mm (IQR = 0.38-1.00 mm) for LMM and 2.80 mm (IQR = 1.75-4.50 mm) for NM. Most SSM and NM were located on the trunk, most LMM on the face, and most ALM on the feet. Ulceration was present in 38.7% of NM, 34.4% of ALM, 7.9% of SSM, and 5.1% of LMM.

**Table 1.** Baseline table of all patients with a single primary cutaneous melanoma in the Netherlands from 2000 to 2014^a^

Characteristic	Total (n = 48 361)	SSM (n = 38 373)	NM (n = 7059)	LMM (n = 2500)	ALM (n = 429)
Subtype, No. (%)					
SSM	38 373 (79.3)	−	−	−	−
NM	7095 (14.6)				
LMM	2500 (5.2)				
ALM	429 (0.9)				
Sex, No. (%)					
Female	27 270 (56.4)	21 978 (57.3)	3605 (51.1)	1415 (56.6)	272 (63.4)
Male	21 091 (43.6)	16 395 (42.7)	3454 (48.9)	1085 (43.4)	157 (36.6)
Age at diagnosis, mean (SD), y	56.39 (16.07)	54.49 (15.44)	61.07 (16.88)	71.09 (12.37)	63.70 (14.82)
18-35	5118 (10.6)	4507 (11.7)	547 (8.1)	22 (0.9)	15 (3.5)
36-55	17 974 (37.2)	15 629 (40.7)	1975 (28.0)	261 (10.4)	109 (25.4)
56-75	18 899 (39.1)	14 583 (38.0)	2919 (41.4)	1193 (47.7)	204 (47.6)
>75	6370 (13.2)	3554 (9.5)	1591 (22.5)	1024 (41.0)	101 (23.5)
Breslow, median (IQR), mm	0.86 (0.50-1.60)	0.76 (0.50-1.20)	2.80 (1.75-4.50)	0.60 (0.38-1.00)	2.02 (1.20-4.00)
0.1-0.7	20 545 (42.5)	18 716 (48.8)	173 (2.5)	1601 (64.0)	55 (12.8)
0.8-1.0	7987 (16.5)	7269 (18.9)	378 (5.4)	309 (12.4)	31 (7.2)
1.1-2.0	10 576 (21.9)	8263 (21.5)	1815 (25.7)	368 (14.7)	130 (30.3)
2.1-4.0	6148 (12.7)	3161 (8.2)	2717 (38.5)	159 (6.4)	111 (25.9)
>4.0	3105 (6.4)	964 (2.5)	1976 (28.0)	63 (2.5)	102 (23.8)
Localization, No. (%)					
Head and neck	5983 (12.4)	3186 (8.3)	1137 (16.1)	1660 (66.4)	0 (0)
Trunk	20 438 (42.3)	17 527 (45.7)	2651 (37.6)	260 (10.4)	0 (0)
Arms	7035 (14.5)	5442 (14.2)	1231 (17.4)	280 (11.2)	82 (19.1)
Legs	13 358 (27.6)	10 969 (28.6)	1805 (25.6)	240 (9.6)	334 (77.9)
Missing	1547 (3.2)	1249 (3.3)	235 (3.3)	60 (2.4)	3 (0.7)
Ulceration, No. (%)					
No	34 480 (71.3)	29 233 (76.2)	3243 (45.9)	1176 (47.0)	228 (53.2)
Yes	6042 (12.5)	3035 (7.9)	2732 (38.7)	128 (5.1)	147 (34.3)
Missing	7839 (16.2)	6105 (15.9)	1084 (15.4)	1196 (47.8)	54 (12.6)
AJCC stage at time of diagnosis, No. (%)					
I	35 442 (73.3)	31 586 (82.3)	1535 (21.7)	2160 (86.4)	161 (37.5)
II	10 618 (22.0)	5347 (13.9)	4727 (67.0)	335 (13.4)	209 (48.7)
III	2301 (4.8)	1140 (3.8)	797 (11.3)	5 (0.2)	59 (13.8)
Deaths, No. (%)	8619 (17.8)	5230 (13.6)	2665 (37.8)	583 (23.3)	141 (32.9)
Follow-up, median (IQR), mo	73.8 (43.5-120.7)	76.9 (45.8-123.9)	62.6 (33.6-114.0)	59.4 (34.4-97.2)	56.6 (34.9-93.2)

a AJCC = American Joint Committee on Cancer; ALM = acral lentiginous melanoma; IQR = interquartile range; LMM = lentiginous malignant melanoma; NM = nodular melanoma; SSM = superficial spreading melanoma.

### Survival Analyses

Before multivariable analysis, we found no linear association between age and survival when assessing linearity for continuous variables. Therefore, age was categorized into 10 equal groups based on the number of events (death). No other violations in proportionality or linearity were found. The hazard ratios and 95% confidence intervals related to subtype were identical when missing ulceration status (16.2%) was regarded as a separate “missing” category or when missing ulceration status was included in the “negative” category (data not shown). In all of the following analyses, we therefore regarded missing ulceration status as negative. A total of 43 872 (90.7%) patients were included in the multivariable analysis. To calculate the main effect of each melanoma subtype, using SSM as a reference, statistically significant hazard ratios for NM (HR = 1.06, 95% = CI 1.01 to 1.12, *P* = .04) and ALM (HR = 1.26, 95% CI = 1.06 to 1.50, *P* = .008) were found. For LMM, no statistically significant difference was found (*P* = .65).

### Effect of Subtype per Breslow Thickness and Ulceration Status

**Figure 1.**
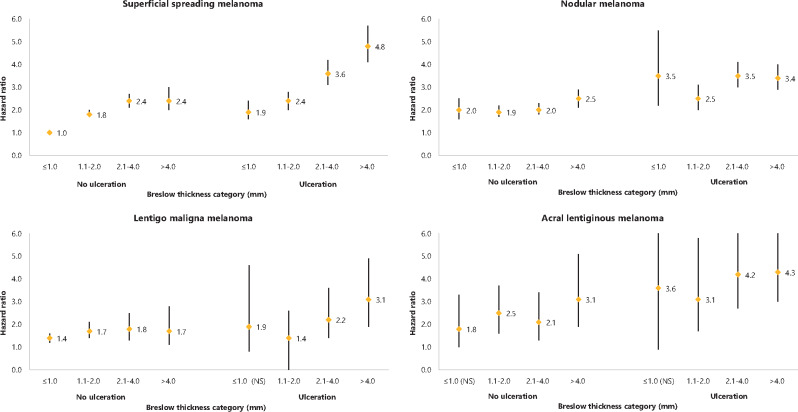
Graphical representation of hazard ratios with 95% confidence interval for each Breslow thickness category, per melanoma subtype and per ulceration status for death from all causes. Superficial spreading melanoma 1.0 mm or less without ulceration is used as a reference category. NS = not statistically significant.

Because we hypothesized that the effect of melanoma subtype was different for tumors with different Breslow thickness, an interaction term of Breslow thickness with melanoma subtype was included in the model. A statistically significant interaction effect between melanoma subtype and Breslow thickness was observed (*P* = .001). The effect of melanoma subtype at different values of Breslow thickness and stratified for ulceration is shown in Figure 1. SSM of 1.0 mm or less without ulceration was used as a reference category for all analyses presented in the different figure panels. Among patients with Breslow thickness 1.0 mm or less and no ulceration, NM showed a 2-fold increased risk (HR = 1.96, 95% CI = 1.58 to 2.45) compared with SSM. Compared with 1.0 mm or less SSM without ulceration, the hazard ratio for 1.0 mm or less SSM with ulceration was 1.94 (95% CI = 1.55 to 2.44), and that for 1.0 mm or less NM with ulceration was 3.46 (95% CI = 2.17 to 5.50). NM patients with tumors greater than 1.0 mm did not show worse survival than SSM patients.

## Discussion

In this study, we showed that NM and ALM melanoma subtypes had worse survival than SSM and LMM subtypes. NM subtype especially affected survival among melanomas that were thin (≤1.0 mm).

Interestingly, there is little literature with sufficient number of patients evaluating the role of melanoma subtype on survival (Table 2 provides an overview, including all variables included in the models). The most recent and largest study was performed by Lattanzi et al. (12), who included 118 508 patients using Surveillance, Epidemiology, and End Results data from 1973 to 2012. They showed that compared with SSM, NM was a statistically significant risk factor for all-cause mortality (HR = 1.55, 95% CI = 1.41 to 1.70). As in our study, stage IV patients were excluded. Other melanoma subtypes besides SSM and NM were not analyzed. Lindholm et al. (11) included 6191 Swedish stage I and II melanoma patients diagnosed with SSM, NM, LMM, or ALM between 1990 and 1999. They observed a hazard ratio for disease-specific-survival of 1.35 (95% CI = 1.08 to 1.70) for NM compared with SSM. LMM and ALM were not found to be independent predictors for mortality. On the contrary, Robsahm et al. (13) did not find melanoma subtype to be an independent predictor for melanoma-specific survival when they analyzed 5010 Norwegian melanoma patients diagnosed between 2008 and 2012. They found a hazard ratio of 1.01 (95% CI = 0.79 to 1.29) for NM and a hazard ratio of 0.93 (95% CI = 0.45 to 1.86) for LMM. Although we found that NM was statistically significantly associated with worse survival, the hazard ratio was only 1.06 (95% CI = 1.01 to 1.12), and its statistical significance might also be affected by the large numbers that this study was based on.

**Table 2.** Overview of hazard ratios of NM and ALM vs SMM in large, previously published studies^a^

Study	No. total (No. of NM and No. of ALM)	HR subtype (95% CI)	No. (%) ≤1.0 mm Breslow thickness	Other variables in Cox analysis	Outcome
Lattanzi et al., 2019 (12)	118 508 (21 399 NM; ALM excluded) No. in Cox not mentioned	NM: 1.55 (1.41 to 1.70) ALM: —	37 596 (31.7)	Breslow thickness, ulceration, age, sex, stage, year of diagnosis	OS
Lindholm et al., 2004 (11)	9515 (1821 NM; 156 ALM) 6191 in Cox	NM: 1.35 (1.08 to 1.70) ALM: 0.91 (0.49 to 1.70)	2933 (47.4)	Breslow thickness, ulceration, age, sex, localization, tumor dimension, Clark level, domicil	MSS
Robsahm et al., 2018 (13)	8087 (1527 NM; 32 ALM) 5010 in Cox	NM: 1.01 (0.79 to 1.29) ALM (merged with 106 ‘other’): 0.67 (0.40 to 1.14)	3745 (46.3)	Breslow thickness, ulceration, age, sex, localization, stage, second primary melanoma	MSS
Dessinioti et al., 2019 (17)	20 132 (5062 NM; ALM excluded) 8370 in Cox (T1)	NM: T1: 2.20 (1.28 to 3.78) T2: 1.23 (0.95 to 1.60) T3: 0.84 (0.69 to 1.03) T4: 0.96 (0.79 to 1.17) ALM: —	9681 (48.1)	Breslow thickness, ulceration, age, sex, center	MSS

a ALM = acral lentiginous melanoma; CI = confidence interval; HR = hazard ratio; MSS = melanoma-specific survival; NM = nodular melanoma; OS = overall survival; SSM = superficial spreading melanoma; — = Not applicable.

Our most interesting finding is that we found higher hazard ratios for death for 1.0 mm or less NM compared with 1.0 mm or less SSM in both ulcerated and nonulcerated melanomas. This might reflect the biological aggressiveness of NM. So in case of timely diagnosis of this melanoma subtype, its Breslow thickness can be misleading, because the tumor seems to behave in a more aggressive way than would be expected on the basis of its Breslow thickness. Our finding is supported by Dessinioti et al. (17), who recently compared melanoma-specific survival of 297 thin (defined as ≤1.0 mm Breslow thickness) NM with 9384 thin SSM. They concluded that thin NM is a high-risk melanoma subtype when adjusted for age, sex, Breslow thickness, ulceration, and center heterogeneity (HR = 2.20, 95% CI = 1.28 to 3.78) (Table 2). The biological aggressiveness of relatively thin NM might also be an explanation for the fact that mortality from NM has not decreased with the years (18), even though the median thickness of NM has decreased (19). Also on a molecular level, NM seems to be a distinct melanoma subtype, because it is more frequently associated with NRAS mutations than SSM (20-22), and it has been shown that this mutation is associated with progressive disease (20).

Our data also show worse survival of ALM than of SSM. Although ALM is a relatively rare melanoma subtype, studies have shown that it is an independent predictor for survival (23,24). Gumaste et al. (23) compared 61 ALMs with 183 non-ALMs and found a hazard ratio of 2.64 (*P* = .001) for melanoma-specific survival for ALMs vs non-ALMs. A potential reason that Lindholm et al. (11) and Robsahm et al. (13) found no statistically significantly worse survival for ALM patients could be due to the relatively small number of patients with ALM subtype in these studies (156 and 32 patients, respectively). A delay in diagnosis, and therefore a worse prognosis, might also be caused by the atypical presentation of this melanoma subtype.

Because melanoma subtyping is of prognostic relevance, accuracy of subtyping in daily practice is important and needs to be reproducible between pathologists. We could find only 1 study on reproducibility of melanoma subtyping, describing a substantial to almost perfect agreement for SSM, NM, LMM, and ALM subtypes as kappa values of 0.73, 0.70, 0.70, and 0.83, respectively, were found (25). Furthermore, in the evolving landscape of adjuvant therapies for melanoma patients (26), the role of NM and ALM subtypes may need to be evaluated for the indication of SLNB and adjuvant therapy.

Our main strength is that we thoroughly assessed the effect of melanoma subtype in different strata of Breslow thickness and ulceration status. Our large sample size allowed us to do this not only for SSM and NM but also for the less prevalent LMM and ALM subtypes. The use of nationwide data resulted in an unselected study population and increased the generalizability of our results. Limitations that go hand in hand with the retrospective nature of our study are missing data. In our study, the missing data were relatively few (9.3%). For our analyses, we regarded missing ulceration status as absent. Although this is an assumption, it is likely to be true for the majority of patients (27). Eigentler et al. (27) used a predictive model for missing ulceration status (n = 7107) in their nationwide study in stage I-III patients (n = 15 158) and estimated 4.9% to be ulcerated. In addition, we have performed a sensitivity analysis including missing ulceration status as a separate “missing” category, which showed no changes in hazard ratios and 95% confidence intervals. Another limitation is that we assumed an SLNB negative outcome in cases where no SLNB was performed. Because SLNB was performed in 44% of patients with a melanoma greater than 1.0 mm Breslow thickness, we might have missed patients who should have been categorized as stage III when SLNB would have been performed and are now categorized as stage II. Because NM and ALM have a higher chance of SLNB positivity, the staging category of these patients might have been underestimated. Although we correct for stage in multivariable analysis, there may thus be some residual confounding effect in NM and ALM patients. A final limitation regarding the analyses is that one could argue that multiple comparisons have been made and that a multiple hypothesis testing correction should have been performed. In that case, our findings would be no longer statistically significant and therefore should be interpreted with care.

All in all, we have shown that melanoma subtype is an independent predictor for survival for melanoma patients, NM and ALM being prognostically worse. NM subtypes especially showed worse survival among melanomas that were thin (≤1.0 mm). Incorporation of histologic subtype into prediction models may lead to better prognostication of melanoma patients.

## Funding

None.

## Notes

**Role of the funder:** Not applicable.

**Disclosures:** The authors declare that they have no conflict of interest.

**Author contributions:** Conception or design of the work, acquisition, analysis, and interpretation of data for the work: All authors. Drafting the work and revising it critically for important intellectual content: All authors. Final approval of the version to be published: All authors. Agreement to be accountable for all aspects of the work in ensuring that questions related to the accuracy or integrity of any part of the work are appropriately investigated and resolved: All authors.

## Data Availability

The data underlying this article were provided by PALGA: the Dutch Pathology Registry. Data will be shared on request to the corresponding author with permission of PALGA.
